# Verses

**Published:** 1906-06

**Authors:** 


### VERSES.

### Dat Ole Crow.

Dat slick black crow
Am a 'ceitful ole case,
Wid er preacher-like cote
An' sollum, sad face.

He hides een de tree
'Till youse dun gone,
Den he caws ter de gang
An' goes fer yer cawn.

Yer can't git ter shoot 'im,
An' he walks roun' yer trap,
Try ebry way ter git 'im,
He don't keer er rap.

He looks like er mourner,
But er leder on er revel
You'd tek 'im fer er saint,
Dat son ob de Devil.

*B. H. Teague.*

### Do It Now.

When you've got a job to do,
Do it now!
If it's one you wish was through,
Do it now;
If you're sure the job's your own,
Don't hem and haw and groan—
Do it now!
Don't put off a bit of work,
Do it now!
It doesn't pay to shirk,
Do it now!
If you want to fill a place
And be useful to the race,
Just get up and take a brace,
Do it now!
Don't linger by the way,
Do it now!
You'll lose if you delay,
Do it now!
If the other fellows wait,
Or postpone until it's late,
You hit up a faster gait—
Do it now!

*Frank Farrington in New York Sun.*

---

### Automobile—Ambulance.

He tried to cross the crowded street—
Must have been in a trance;
Chug, chug, the automobile came—
Clang, clang, the ambulance.

*Buffalo Express.*

---

### Man Only Forgets.

Man—only man—forgets his due
To God, who is both life and food;
Whose law is just, whose word is true—
Who "out of evil bringeth good."

*Josephine Pope.*

### The Good Man.

He lived for those who loved him,
For those who knew him true;
For the heaven bright above him,
And the good that he might do.

For the cause that needed assistance,
For the wrongs that lacked resistance,
For the future in the distance,
And the good that he might do.

*Exchange.*

